# *Artemisinin* Loaded Cerium-Doped Nanopowders Improved In Vitro the Biomineralization in Human Periodontal Ligament Cells

**DOI:** 10.3390/pharmaceutics15020655

**Published:** 2023-02-15

**Authors:** Ioannis Tsamesidis, Anna Theocharidou, Anastasia Beketova, Maria Bousnaki, Iason Chatzimentor, Georgia K. Pouroutzidou, Dimitrios Gkiliopoulos, Eleana Kontonasaki

**Affiliations:** 1 Department of Prosthodontics, School of Dentistry, Faculty of Health Sciences, Aristotle University of Thessaloniki, GR-54124 Thessaloniki, Greece; 2 Laboratory of Advanced Materials and Devices (AMDeLab), School of Physics, Faculty of Sciences, Aristotle University of Thessaloniki, GR-54124 Thessaloniki, Greece; 3 Laboratory of Chemical and Environmental Technology, Department of Chemistry, Aristotle University of Thessaloniki, GR-54124 Thessaloniki, Greece

**Keywords:** artemisinin, bio mineralization, cerium nanopowders, human periodontal ligament cells, bone regeneration

## Abstract

Background: A promising strategy to enhance bone regeneration is the use of bioactive materials doped with metallic ions with therapeutic effects and their combination with active substances and/or drugs. The aim of the present study was to investigate the osteogenic capacity of human periodontal ligament cells (hPDLCs) in culture with artemisinin (ART)-loaded Ce-doped calcium silicate nanopowders (NPs); Methods: Mesoporous silica, calcium-doped and calcium/cerium-doped silicate NPs were synthesized via a surfactant-assisted cooperative self-assembly process. Human periodontal ligament cells (hPDLCs) were isolated and tested for their osteogenic differentiation in the presence of ART-loaded and unloaded NPs through alkaline phosphatase (ALP) activity and Alizarine red S staining, while their antioxidant capacity was also evaluated; Results: ART promoted further the osteogenic differentiation of hPDLCs in the presence of Ce-doped NPs. Higher amounts of Ce in the ART-loaded NPs inversely affected the mineral deposition process by the hPDLCs. ART and Ce in the NPs have a synergistic role controlling the redox status and reducing ROS production from the hPDLCs; Conclusions: By monitoring the Ce amount and ART concentration, mesoporous NPs with optimum properties can be developed towards bone tissue regeneration demonstrating also potential application in periodontal tissue regeneration strategies.

## 1. Introduction

Periodontitis is an inflammatory disease of the gums and tooth surrounding structures which affects 20–50% of the adult population worldwide [1]. Severe periodontitis is associated with the destruction of periodontal tissues, including periodontal ligament, alveolar bone and cementum, eventually leading to the tooth loss. Over the past years, major scientific advances in periodontology have introduced innovative treatments for periodontal diseases; however, there is still no ideal therapeutic approach to regenerate periodontal bone defects. In addressing these issues, regenerative therapies using genes, stem cells, growth factors, physical and pharmacological factors in combination with drug delivery agents have been exploited recently. Among them, local delivery of ion-containing (Ca, Si) bioactive nanocarriers with controlled and sustained drug release seems to be a very promising strategy to induce osteogenesis [2]. Mesoporous materials have been developed for the delivery of ions and drugs with applications in dentistry. It has been reported that calcium and cerium doping can have a positive effect on the proliferation of human periodontal ligament fibroblasts [3].

Artemisinin (ART) is a sesquiterpene lactone compound with an endoperoxide bridge, originally derived from the sweet wormwood (Artemisia Annua) used in combination therapy with quinoline drugs for malaria treatment and proposed with other partner drugs too [4]. Nowadays there are several derivatives of this drug with enhanced water solubility and improved pharmacokinetics, including dihydroartemisinin (DHA), artesunate, artemisinic acid, artemether and arteether available in the form of injections or oral tablets [5]. Artemisinin and its derivatives have a wide range of other clinical applications and documented pharmaceutical effects for treatment of bacterial, fungal, protozoal and parasite-related infections, cancer, atherosclerosis, diabetes mellitus, osteoporosis and fibrosis [6,7]. In addition, it has anti-inflammatory, immunomodulatory and antioxidant activities that have been reported elsewhere [8]. The pharmacologic action of artemisinin is based on an endoperoxide bridge, which is in the form of a 1,2,4-trioxane ring, which is broken in the presence of Fe^2+^, leading to the generation of free radicals and reactive oxygen species (ROS). In turn, ROS initiate a cascade of cytostatic and cytotoxic effects in target cells which have high levels of intracellular iron, such as malaria parasites [4], various cancer cells, and osteoclasts [9]. According to the current knowledge, artemisinin and its derivatives (Artesunate, DHA, Artemether) do not show serious side effects in human clinical studies in the prescribed doses when administrated for malaria treatment, while long-term availability in elevated doses can cause cardiotoxicity, genotoxicity and embryotoxicity in animal studies and thus must be carefully monitored [10]. However, for the local release of ART and its derivatives from scaffolds, nanoparticles and other bone substitute materials, there is no available data. At the same time, artemisinin is a quite cheap and easy to be found natural material.

Recent studies have shown that ART’s compounds are promising drugs for the effective treatment of osteoclast-related bone diseases, such as osteoporosis, osteoarthritis, Paget’s disease and cancer-induced osteolysis. The essential role of artemisinin derivatives against bone resorption was demonstrated in several animal models, including ovariectomized mice osteoporosis models [11,12], lipopolysaccharide (LPS) and Ti-particle induced osteolysis mouse model [13,14] and osteoarthritis-induced bone loss model [15]. Artemisinin compounds suppress the receptor activator of nuclear factor-κ B ligand (RANKL) induced osteoclast differentiation via multiple signaling pathways, thereby inhibiting bone resorption [9]. Moreover, osteoprotective and osteostimulating effects of ART were reported in different progenitor cells, such as human mesenchymal stem cells (HMSCs) [16] and dental pulp stem cells (DPSC) [17], indicating that this drug might be applicable for fracture treatment and tissue engineering. Hu et al. demonstrated that artemisinin restored the suppressed osteogenic differentiation of human DPSCs under inflammation and hypoxia conditions through activation of the Wnt/β-catenin signaling pathway and upregulation of CA9 expression in DPSCs [17]. Fang et al., reported that artemisinin protected bone marrow-derived mesenchymal stem cells (BMSCs) from oxidative stress injury in a dose-dependent manner by activating the c-Raf-Erk1/2-p90rsk-CREB signaling pathway [18]. 

Moreover, artemisinin derivatives present a synergetic effect with other drugs in malaria [4] as well as ions and ion-containing nanoparticles [19]. In this respect, poly(norepinephrine)-coated FeOOH nanocarriers [20] and Fe-containing mesoporous nanoparticles [21,22] have been produced for simultaneous artemisinin delivery and transport of Fe ions to the targeted tumor. Due to the increased Fe concentration in tumor microenvironment and internalization of nanoparticles by the tumor cells, the efficiency of such combination therapy was significantly enhanced compared with free ART [21]. High anticancer activity and synergetic effect of ART of Mn ^2+^ from Fe_3_O_4_@MnSiO_3_-FA nanospheres was demonstrated in vivo [23]. Furthermore, Avitabile et al. reported the enhanced antimalarial activity of bio-capped silver nanoparticles from Artemisia species [24].

In the context of bone regeneration, artemisinin loaded mesoporous cerium-doped calcium silicate nanocarriers have been developed recently [3]. The nanoparticles appeared to be highly bioactive and biocompatible, due to the presence of Ca and Si ions in their composition, which are also present in the mineralized bone matrix. Moreover, Ce also exists in healthy bones in microquantities and accumulates with age. Ce ions are also known for their anti-oxidation, anti-inflammation and angiogenic activities and, thus, could promote bone regeneration [25]. Although the beneficial effect of ART-containing Si-Ca-Ce mesoporous NPs on periodontal ligament cell viability has been reported [3], its osteogenic potential, as well as its molecular mechanism of interaction between inorganic ions and ART has not been investigated so far. Based on the aforementioned, the aim of this study was to investigate the potential of ART-containing Si-Ca-Ce mesoporous NPs to induce osteogenic differentiation of human periodontal ligament cells. 

## 2. Materials and Methods

### 2.1. Characterization of NPs

The synthesis of five different mesoporous NPs was assessed via a surfactant-assisted cooperative self-assembly process (Table 1). Mesoporous SiO_2_ (Si-NP), Ca-doped silica (SiCa-NP) and three different Ca/Ce co-doped silicate NPs (SiCaCe-NPs) were synthesized using a basic environment (with pH values between 12.0 and 12.5) and characterized, as previously described by our team with the following techniques: scanning electron microscopy (SEM), X-ray fluorescence spectroscopy (XRF), Fourier transform infrared spectroscopy (FT-IR), X-ray diffraction analysis (XRD) and N_2_ porosimetry [3]. The tested groups were the ART-loaded NPs and the control group were the unloaded NPs. ART (1 mM) was dissolved in dimethyl sulfoxide (DMSO) and PBS. Nanoparticles were added at a concentration of 1 mg/mL to that solution, and stirred (300 rpm) at room temperature in dark conditions for 24 h. The ART-loaded NPs were centrifuged at 5000× *g* for 15 min, collected and dried at room temperature. The ART loading capacity was further confirmed with the FTIR technique described below.

### 2.2. Fourier Transform Infrared Spectroscopy (FTIR)

The synthesized mesoporous nanomaterials were studied with the use of Fourier transform infrared spectroscopy (FTIR). A PerkinElmer Inc., Waltham, MA, USA spectrometer was used to assess the FTIR measurements that were performed in transmittance mode (400–4000 cm^−1^, 2 cm^−1^ resolution, and 32 scans). For each measurement, a KBr pellet was produced under a pressure of 7 tons, using a ratio of material powder-to-KBr 1:100.

### 2.3. Apatite-Forming Ability in c-SBF

The Ca-containing mesoporous nanomaterials before and after ART-loading were immersed in c-SBF solution using a 1.5 mg/mL ratio sample to solution and were incubated at 37 °C, under renewal conditions [26,27,28] All samples were collected after 24 h of immersion and dried before the FTIR analyses.

### 2.4. Biological Evaluation of the NPs

Characterization of PDLCs and Osteogenic Differentiation Isolation of Human Periodontal Ligament Cells (hPDLCs)

The cell cultures of the human periodontal ligament cells were obtained using human biopsies of periodontal ligament tissues of healthy donors taken during routine third molar extraction and cultured, as previously described [3]. The study was approved by the institutional ethical committee (#110/10-2-2021).

Osteogenic Differentiation

Artemisinin (ART) unloaded and loaded Si, SiCa, SiCaCe1, SiCaCe2.5 and SiCaCe5 nanopowders (NPs) at different concentrations (C1: 12,5 and C2: 125 μg/mL) were sterilized with UV light for 30 min before seeding with the hPDLCs. The cells were seeded at 4 × 10^4^ onto 12-well plates 24 h before to initiate the experiment. A specific osteoinductive medium (OM) was used for the differentiation of hPDLCs. The protocol for the preparation of the OM was presented in detail in previous work [29]. The experimental groups created consisted of: (1) cell-seeded NPs cultured in OM; (2) a control group of cell-seeded NPs cultured in conventional medium-CCM); (3) cells seeded without NPs, cultured in OM as a positive control for induction; and (4) cells without NPs cultured in CCM. The differentiation experiments were performed for 14 and 21 days, and both OM and CCM were changed every second day. The hPDLC’s osteogenic performance was evaluated with two techniques: alkaline phosphatase (ALP) activity and alizarin red staining. Furthermore, the effect of the NP incubation, with the highest concentration (C2), antioxidant capacity was assessed by determining the total antioxidant capacity, as described below.

Alizarine Red S Staining (ARS)

Τhe experimental procedure was initiated with the seeding of the hPDLCs into 12-well tissue culture plates at a density of 4 × 10^4^ cells/well and incubated at 37 °C in 5% CO_2_ humidified. The media were then replaced by either CCM alone or with OM combined with one of the selected nanomaterials for 14 or 21 days. The same set of experiments were performed without hPDLCs, only in presence of the NPs, in order to subtract the OD values received from the NPs alone, without cells. The formation of mineralized matrix nodules was detected by alizarin red S staining (Sigma-Aldrich, St. Louis, MO, USA) following standard protocol, as previously described [29]. Orange-red staining in well plates indicates the formation of mineralized matrix nodules in the extracellular matrix that were visualized through an inverted microscope. The measurement of the ARS staining levels was performed by elution with 10% (*w/v*) cetylpyridinium chloride at room temperature for 20 min. OD was measured by a microplate spectrophotometer at 540 nm.

Alkaline Phosphatase Activity

The levels of ALP after 14 and 21 days of hPDLCs cultured with ART loaded and unloaded Si, SiCa, SiCaCe1, SiCaCe2.5 and SiCaCe5-NPs, in both concentrations (C1 and C2) were evaluated using CCM or OM by analyzing both cell lysates and their supernatants. All conditions were compared to the ALP activity detected in the hPDLCs cultured without NPs in the same media. Once the experiment was performed, all of the samples (cell lysates or supernatants) were mixed gently with a specific ALP alkaline buffer solution (Apollo Scientific—BI4545 and BIN0446), as previously described [29]. The P-nitrophenylphosphate amount was calculated by measuring the absorbance of 405 nm.

### 2.5. Antioxidant Capacity

The cell lysates and the supernatants from the ALP and alizarin experiments with the highest concentration of NPs (C1) were also used to evaluate the total antioxidant capacity (TAC). TAC was obtained using a TAC kit (TAC colorimetric assay kit, Cayman Chemical Co, USA) with the method of TEAC, as previously described [30]. The antioxidant capacities (TEAC) of the samples are expressed as mmol/L/viable cells using a calibration curve plotted with different amounts of Trolox, and their absorbance measured at 405 nm. The data are the results from three independent experiments.

### 2.6. Statistical Analysis

All of the results are presented as the mean and standard deviation (SD) of at least three independent experiments in sextuplicate A Student’s *t*-test was used for testing the differences between groups with the statistical significance set at a = 0.05. 

## 3. Results

### 3.1. Characterization of the NPs

#### 3.1.1. ART-Loading Capacity

**Figure 1:**
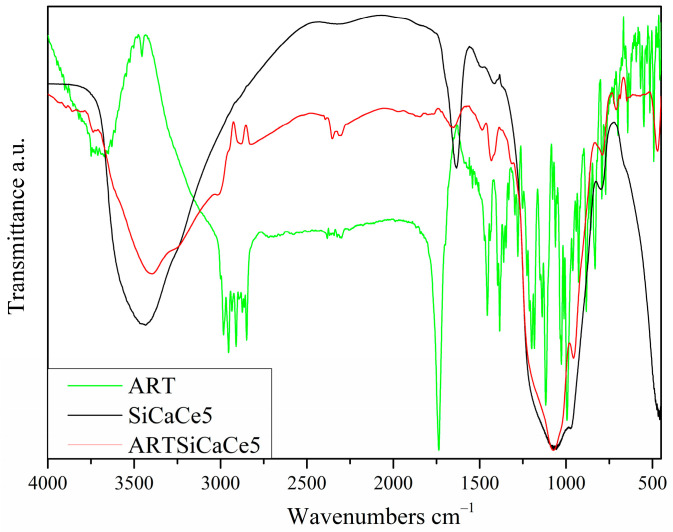
Representative FTIR spectra of SiCaCe5 before and after loading with ART.

The confirmation of the ART encapsulation was performed by a FTIR analysis. A representative spectrum of ART-loaded samples with the spectrum of ART as a reference are presented in Figure 1.

The shoulder at around 960 cm^−1^ corresponds to the stretching vibration of the Si–O- Ca bond. The broad band between 900 and 1200 cm^−1^ is correlated to the asymmetric stretching vibrations of the Si–O–Si bonds. Moreover, the band at 795 cm^−1^ is attributed to the Si–O–Si bending vibrations. The broad peak between 3670 and 3070 cm^−1^ is attributed to O-H groups of absorbed water, while the peak at 1637 cm^−1^ is due to the vibration of Si–OH bond and water molecules. Finally, the peaks at around 1418 and 1495 cm^−1^ are related to the C–O bond vibration [3,31,32].

The spectrum of pure ART presents the peaks at 996, 926 and 832 cm^−1^ that correspond to C-C stretching vibrations. The peaks at 1026 and 1012 cm^−1^ are attributed to -C-O-stretching vibrations, the peaks at around 1116 and 1384 cm^−1^ are related to -O- and -CH_3_ stretching vibrations, respectively. Moreover, the peak at around 1456 cm^−1^ is attributed to -CH_2_ bending vibrations, the peak at 1738 cm^−1^ is presented due to the C=O stretching vibrations, the peaks at 2800 cm^−1^ and 3020 cm^−1^ are related to -CH_2_ stretching vibrations, and the O-H vibrations are confirmed by the presence of the peak at around 3710 cm^−1^ [21,31].

The presence of some characteristic peaks corresponding to ART in the spectrum of the ART-loaded mesoporous NPs verifies the presence of ART in the NPs, although some ART peaks may be overlapped at around 900–1200 cm^−1^ [33]. For SiCaCe5 loaded with ART (sample ARTSiCaCe5), the new peaks at around 3718, 3010, 2894, 1782, 703, 695, 646 and 556 cm^−1^, as well as the shifting of the peak at around 1428 cm^−1^, confirm the incorporation of ART into the mesopores of mesoporous nanomaterials.

Moreover, after the ART-loading, there is a presence of a double peak at around 600–555 cm^−1^ at the spectrum of the ARTSiCa, which is related to the bending vibration of the P–O bond. This finding, combined with the sharpening of the broad peak at about 900–1200 cm^−1^, is due to the bending vibration of the (PO_4_)^3−^ group, the shoulder at 960 cm^−1^, which can be attributed to the (PO_4_): PO stretching vibration, can be justified due to the formation of hydroxyapatite after 24 h of stirring into the PBS-ART solution.

#### 3.1.2. Apatite-Forming Ability in c-SBF

**Figure 2:**
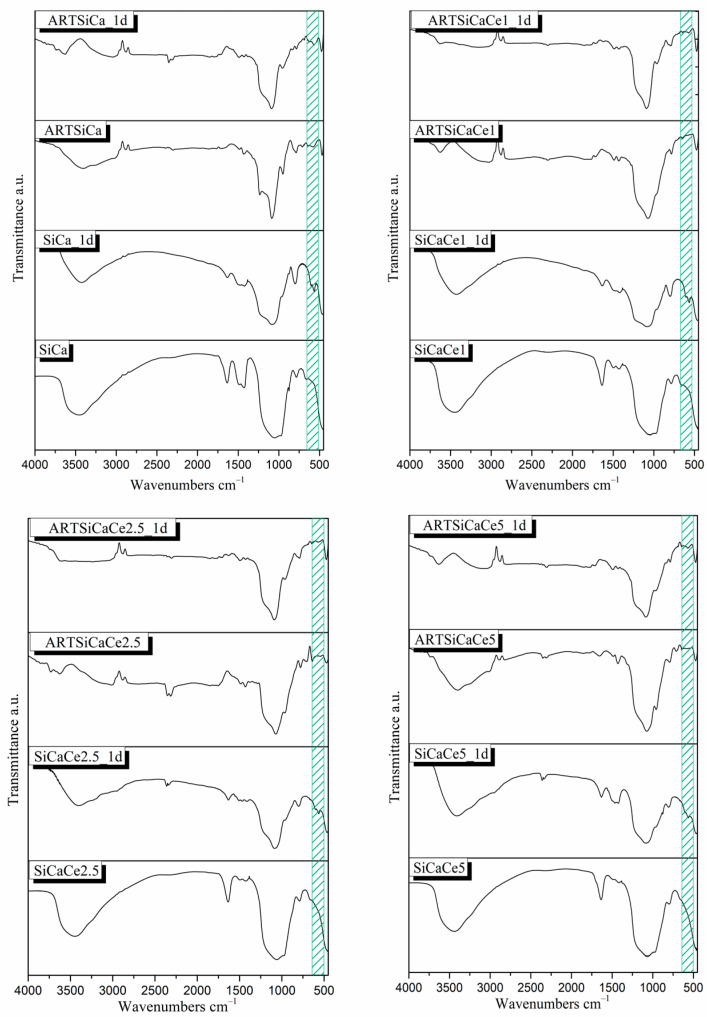
FTIR spectra of synthesized samples before and after ART-loading and before and after the immersion in SBF.

The FTIR spectra of all NPs after the immersion in simulated body fluid for 24 h are presented in Figure 2. Following 24 h of immersion, the FTIR spectra of all samples before ART-loading did not present any remarkable differences. All studied materials presented the characteristic peaks that confirm Ca-P or hydroxyapatite (HAp) formation on the surface of the NPs. More specifically, the sharpening of the wide peak between 900 and 1200 cm^−1^ corresponds to the bending vibration of the (PO_4_)^3−^ group, as well as the double peak at around 608–600 and 580–555 cm^−1^, corresponding to the bending vibration of the P–O bond of Hap, and the presence of the weak shoulder at around 960 cm^−1^ is attributed to (PO_4_): PO stretching vibration. Moreover, the sharpening of the peak at 795 cm^−1^ is attributed to the stretching vibration of the Si–O–Si bond probably due to the polycondensation of silanols [3,29,32,34].

The reduced intensity of the double peak around 608–600 and 580–555 cm^−1^ corresponding to the P–O bending vibration in the spectra of ART-loaded samples after 24 h of immersion might be an indication of amorphous calcium phosphate rather than completely crystalized HAp. Due to the limitations of the FTIR, XRD or other complementary techniques would clarify further the exact nature of the developed calcium-phosphate phases [35]. 

### 3.2. Alizarin Red S Staining of the hPDLCs Cultured with or without NPs 

**Figure 3:**
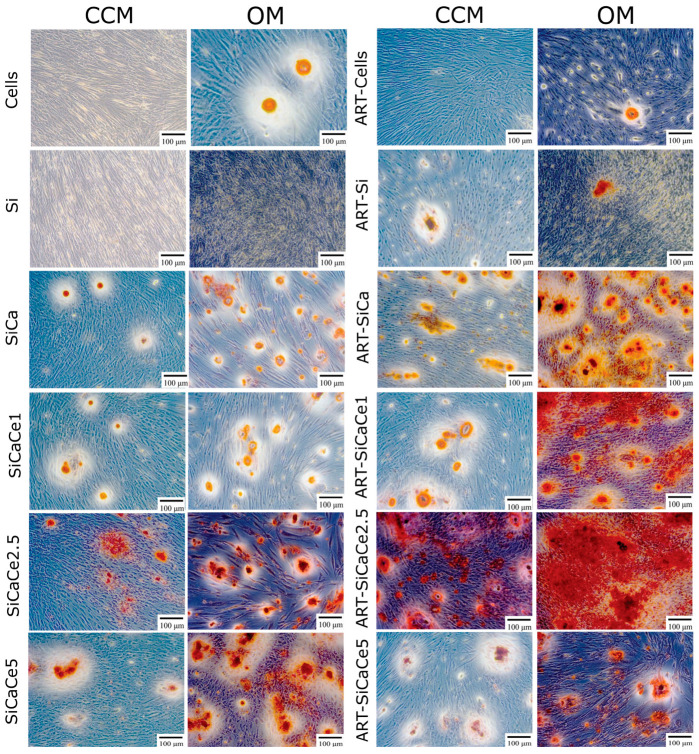
Photomicrographs of the in vitro deposition of calcium of hPDLCs alone or cultured with the ART-loaded and unloaded NPs, at the highest tested concentration (C2) (125 mg/mL) and two different media (CCM, OM).

Alizarin red S staining assay was performed to investigate the mineralized matrix nodules which represent osteoblast-phenotypic markers of a successful osteogenic differentiation in hPDLCs, when incubated with the ART-loaded and unloaded cerium-doped NPs. Figure 3 presents the increase of deposition of calcified materials after 21 days of incubation and the NPs containing Ca and Ce presented remarkable mineralization, especially at this time-point in OM. Si-NPs, served as negative controls, without promoting the in vitro deposition of calcium in hPDLCs, even in OM.

**Figure 4:**
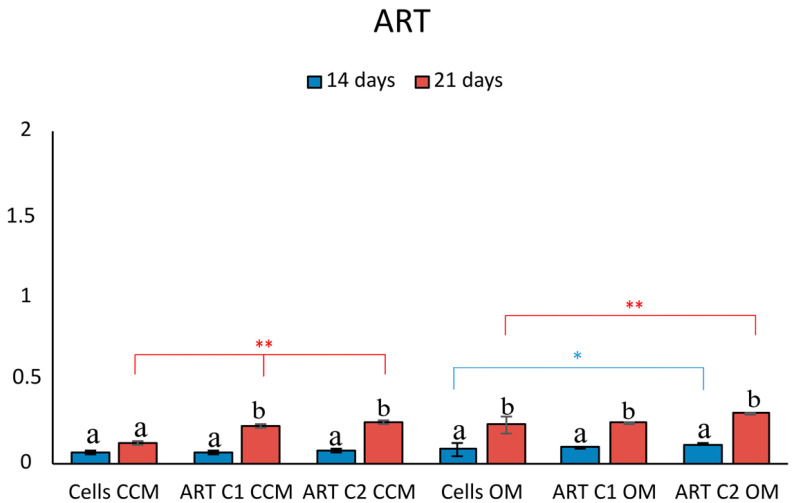
Quantitative analysis of ARS in cells cultured in both media (CCM and OM) in the presence of ART. Bars indicate statistically significant differences between cells and cells incubated with different concentrations of ART * = *p* < 0.05, ** = *p* < 0.01, while different letters suggest statistically significant differences (*p* < 0.001) between the two time points.

When cells were cultured in the presence of ART, there was no significant difference in their biomineralization capacity (Figure 4). 

**Figure 5:**
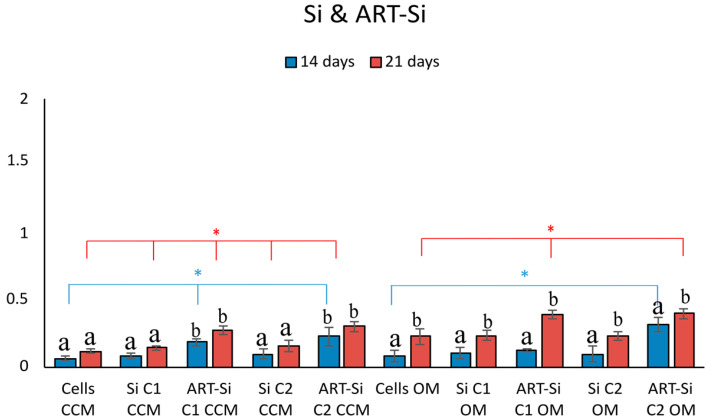
Quantitative analysis of ARS in cells cultured in both media (CCM and OM) in the presence of ART-loaded and unloaded SiNPs. Bars indicate statistically significant differences between cells and cells incubated with different concentrations of NPs * = *p* < 0.05, while different letters suggest statistically significant differences (*p* < 0.001) between the two time points.

When Si-NPs were loaded with ART, a statistically significant increase was observed (*p* * < 0.05) compared to cells alone, indicating a beneficial effect of ART on the hPDLC’s in vitro deposition of calcium (Figure 5). 

**Figure 6:**
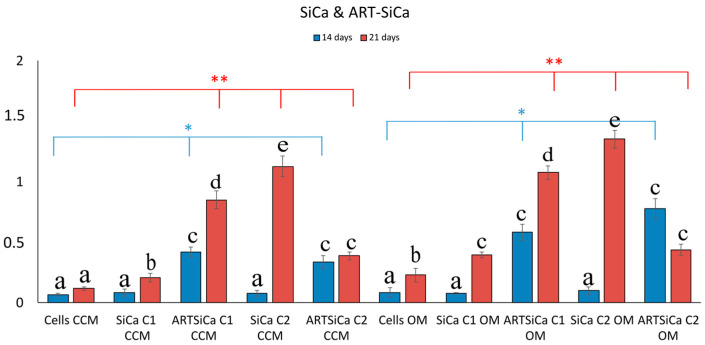
Quantitative analysis of ARS in cells cultured in both media (CCM and OM) in the presence of ART-loaded and unloaded SiCaNPs. Bars indicate statistically significant differences between cells and cells incubated with different concentrations of NPs * = *p* < 0.05, ** = *p* < 0.01, while different letters suggest statistically significant differences (*p* < 0.001) between the two time points.

Si-NPs doped with calcium [SiCa-NPs] presented a delayed, yet significant increase in OD values after 21 days of cell culture (Figure 6). However, this increase was more pronounced in ART-SiCa-NPs even at 14 days. It should be noted that ART did not suppress the in vitro biomineralization of SiCa-NPs, but instead, the deposition of calcium salts was enhanced 2–6 fold (*p* ** < 0.01) compared to the cells alone (control +OM). A clear and statistically significant increase (*p* < 0.05) was recorded for the cells treated with SiCa + OM without ART and in ART-SiCa + OM at the highest tested concentration (Figure 3 and Figure 6).

**Figure 7:**
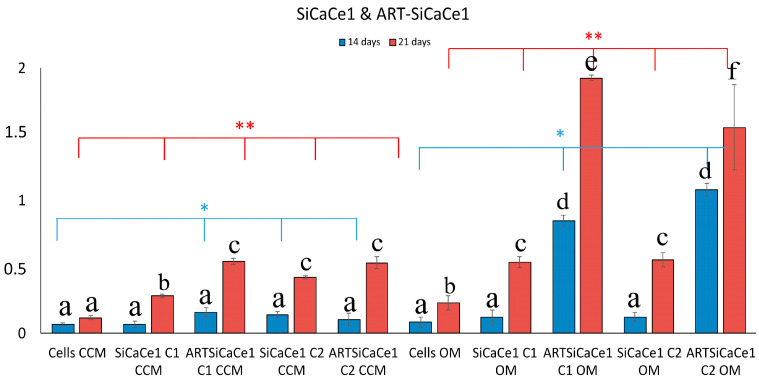
Quantitative analysis of ARS in cells cultured in both media (CCM and OM) in the presence of ART-loaded and unloaded SiCaCe1NPs. Bars indicate statistically significant differences between cells and cells incubated with different concentrations of NPs * = *p* < 0.05, ** = *p* < 0.01, while different letters suggest statistically significant differences (*p* < 0.001) between the two time points.

Regarding the different amounts of cerium doping (1, 2.5 and 5%) and their bio mineralization effect, a dose dependent behavior was observed in terms of cerium doping. In detail, SiCaCe1-NPs (Figure 7) presented a significant (p * < 0.05) 2-to-4-fold increase of OD values after 21 days of incubation compared to cells, and this behavior was statistically significantly enhanced when the NPs were loaded with ART.

**Figure 8:**
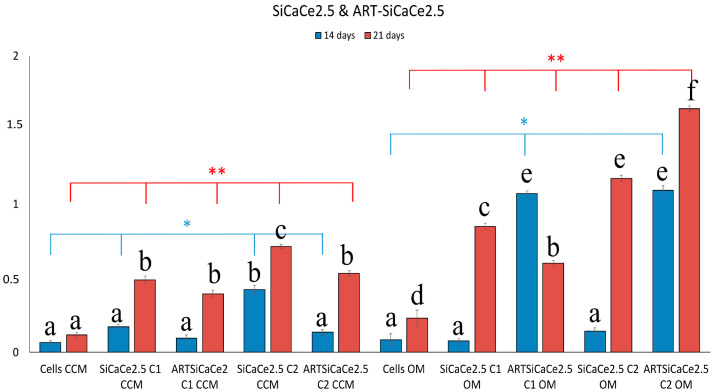
Quantitative analysis of ARS in cells cultured in both media (CCM and OM) in the presence of ART-loaded and unloaded SiCaCe2.5NPs. Bars indicate statistically significant differences between cells and cells incubated with different concentrations of NPs * = *p* < 0.05, ** = *p* < 0.01, while different letters suggest statistically significant differences (*p* < 0.001) between the two time points.

As shown in Figure 8, SiCaCe2.5-NPs presented a similar or slightly increased bio mineralization compared to cells after 14 days and significantly increased after 21 days (1–3 fold in CCM and 8–10 in OM) (*p* < 0.05), however after ART loading and at the highest NPs concentration (C2), this increase was less compared to ART-loaded NPs at the lowest concentration (C1). The osteogenic differentiation in hPDLCs was superior when the NPs were loaded with ART, but not as much as in presence of ART-SiCaCe 1% of cerium (3 fold increase in OM).

**Figure 9:**
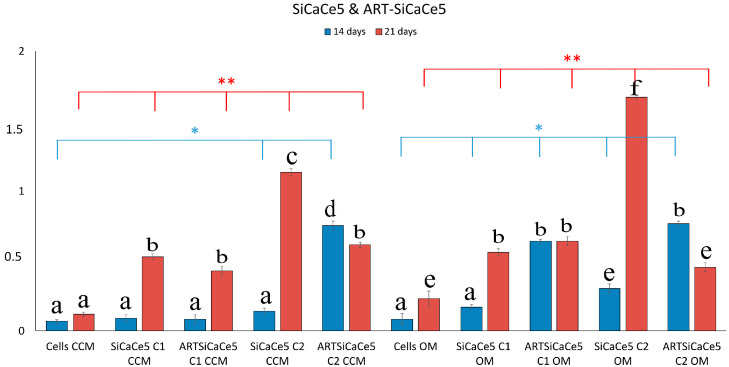
Quantitative analysis of ARS in cells cultured in both media (CCM and OM) in the presence of ART-loaded and unloaded SiCaCe5NPs. Bars indicate statistically significant differences between cells and cells incubated with different concentrations of NPs * = *p* < 0.05, ** = *p* < 0.01, while different letters suggest statistically significant differences (*p* < 0.001) between the two time points.

SiCaCe5 doped with 5% of cerium presented the highest amounts of mineralized matrix nodules compared to the rest of the unloaded NPs (Figure 9). However, when SiCaCe5-NPs were treated with ART, the bio mineralization effect was statistically significantly decreased (p ** < 0.01) compared to the rest of the ART-loaded NPs, indicating an inversely proportional relationship between cerium ions and artemisinin activation. Of note, hPDLCs morphology was changed to polygonal from the original and typical spindle-like shape after 21 days of culture in the OM when cultured with NPs.

### 3.3. Alkaline Phosphatase Activity

The levels of ALP after 14 and 21 days of hPDLCs cultured with unloaded and ART-loaded Si, SiCa, SiCaCe1, SiCaCe2.5 and SiCaCe5-NPs were evaluated using CCM and OM, and an analysis was performed on both cell lysates and supernatant media. 

**Figure 10:**
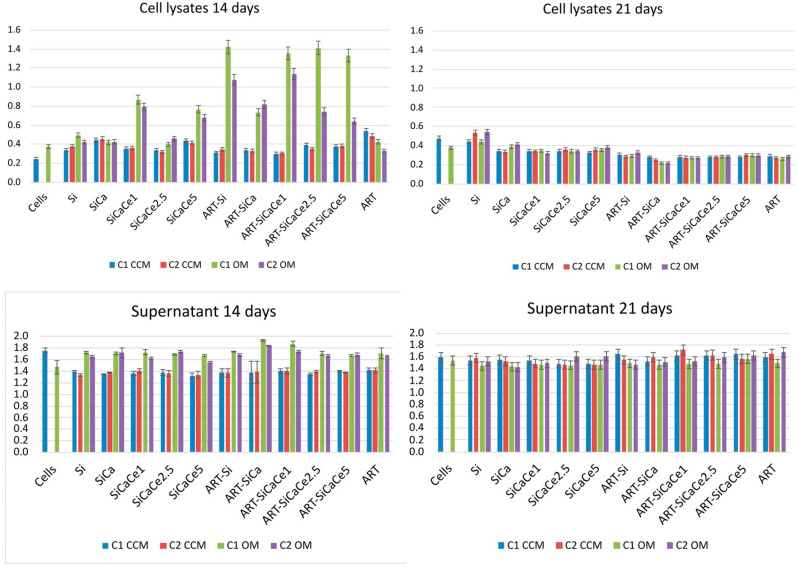
In vitro ALP expression of hPDLCs cultured with unloaded and ART-loaded Si, SiCa, SiCaCe1, SiCaCe2.5 and SiCaCe5-NPs in CCM and OS medium for 14 and 21 days of incubation. Top: ALP levels from cell lysates; bottom: ALP levels from supernatants. Bars indicate statistically significant differences between cells and cells incubated with the NPs.

Figure 10 presents the ALP levels of hPDLCs lysates after 14 and 21 days of incubation. The levels of ALP activity in unloaded NPs do not present any significant differences except for SiCaCe1 and SiCaCe5, where a significant increase is observed in comparison with the negative differentiation control after 14 days of incubation. Our data reveal statistically significantly higher ALP values of all the ART-loaded NPs after 14 days of incubation, compared to the hPDLCs cells without NPs incubation. ART-loaded Si, SiCaCe1, SiCaCe2.5 and SiCaCe5-NPs presented the highest ALP values among all NPs and compared to the +OM control (*p* < 0.05). ALP values were lower at the supernatants of 21 days compared to those of 14 days, but there were no significant differences among the loaded and unloaded NPs. 

### 3.4. Antioxidant Capacity

**Figure 11:**
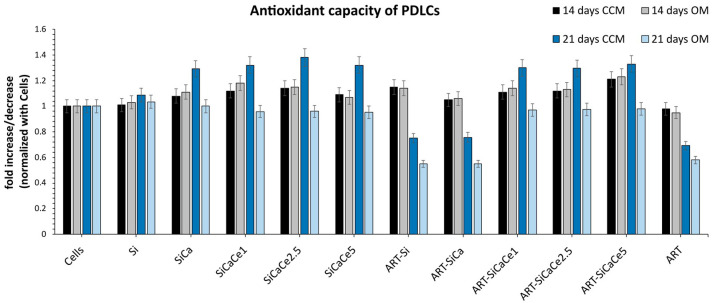
Total antioxidant capacity of hPDLCs after culture with unloaded and ART-loaded Si, SiCa, SiCaCe1, SiCaCe2.5 and SiCaCe5-NPs and the tested NPs at the highest tested concentration (C2). The results are expressed in mM and presented in fold modifications compared to control cells (without NP incubation).

Total antioxidant capacity (TAC) was measured at all tested conditions. Figure 11 presents the fold increase or decrease of hPDLCs after incubation with the NPs at the highest tested concentration (C2) in CCM or OM, normalized with the cells without materials.

In detail, the TAC assay revealed that the presence of NPs did not affect the antioxidant capacity of hPDLCs after 14 days of incubation. The most profound differences were observed after 21 days of incubation with the NPs. In detail, ART alone both in CCM and OM decreases the antioxidant levels of hPDLCs. The same trend decreasing the antioxidant capacity was also observed when hPDLCs were incubated with the ART-loaded NPs in OM (ART-Si, ART-SiCa, ART-SiCaCe1) (*p* < 0.05) except SiCaCe2.5 and SiCaCe5. However, this trend was not observed with the unloaded NPs in OM. When hPDLCs were incubated in CCM with the unloaded, doped NPs, the antioxidant capacity was statistically significantly increased (*p* < 0.01), by approximately 1.5 folds. 

## 4. Discussion

To the best of the authors knowledge, this is the first study to assess the potential of cerium-doped mesoporous NPs with or without the presence of ART to induce bio mineralization via the osteogenic differentiation of hPDLCs cells. Our previous study showed that the ART-loaded mesoporous cerium-doped calcium silicate NPs possess ROS scavenging properties, and proved to be biocompatible, significantly promoting hPDLC proliferation [3]. Here, it is further demonstrated that ART enhanced the deposition of calcium salts in the presence of SiCa-NPs and SiCaCe-NPs. However, an inversely proportional relationship between cerium ions and artemisinin activation was observed regarding calcium deposition and HA formation. Regarding the different amounts of cerium doping (1, 2.5 and 5%) and their bio mineralization effect, a dose dependent behavior was observed, with the cerium-doped NPs without ART with the highest Ce percentage presenting the more pronounced mineralized matrix nodules formation. The effect of different NP concentrations on the osteogenic potential of hPDLCs was assessed. Depending on the experimental group, concentration had a varying effect on calcium deposition. In SiCa NPs, the increasing concentration resulted in increased calcium deposition at 21 days, whereas ART-loaded SiCa NPs led to opposite results. Regarding Ce-doped SiCa NPs, in SiCaCe2.5 and SiCaCe5 NPs, there was a trend towards increased calcium deposition after 21 days in culture with increasing concentration.

Our results are supported by the recent studies which show that cerium-oxide (Ce) NPs can promote osteogenesis and bone mineralization [36,37,38,39,40,41]. More specifically, Wei et al. showed that Ce-NPs stimulate osteogenic differentiation of MSCs along with the induction of macrophage differentiation towards M2-phenotypes [36], exhibiting immunoregulatory properties, while Ce-NPs embedment into composite scaffold can aid bone repair [37]. The mechanism of action through which Ce-NPs affect osteogenesis has not been clarified yet, however different pathways have been proposed and highlighted in the literature. Ce-NPs have demonstrated exceptional scavenging capacity of reactive oxygen species (ROS), comparable to the enzymes (enzyme-mimetic activity), due to their ability to mimic glutathione peroxidase and catalase during the transition of Ce^4+^ to Ce^3+^ [36], and exhibit superoxide dismutase (SOD)-like activity during the Ce^3+^ to Ce^4+^ conversion [37]. Excessive ROS production and increased oxidative stress can lead to osteoblast and osteocyte death, suppressed osteogenesis and diminished bone marrow MSCs differentiation [42]. Nonetheless, Ce-NPs bioactivity and ability to reduce ROS should not be solely attributed to its redox states, but can also be related to components of Ce-NPs surface chemistry, such as particle morphology and surface modification [43]. Luo et al. demonstrated that Ce-NPs promoted the differentiation of precursor osteoblasts by enabling the nuclear translocation of β-catenin protein and activation of the Wnt pathway [44]. Lu et al. showed that incorporation of Ce-NPs in hollow mesoporous bioglass scaffolds activates the ERK pathway, thus stimulating the osteogenic differentiation of bone marrow MSCs [45].

The presence of ART in the culture of hPDL cells promoted the osteogenic differentiation in almost all conditions assessed, as it is depicted by the bio mineralization process and the ALP activity. More specifically, the ART presence in the NPs led to a significant increase in the detection of ALP in cell lysates OM after 14 days of culture at the low concentration of NPs. ALP is an early marker of mineralization, for which expression decreases as the mineralization proceeds [46]. The early induction of differentiation of hPDLCs cultured with the ART-loaded SiCaCe NPs, as indicated by the increased ALP activity in day 14, and is followed by a decrease in ALP activity after 21 days in culture, during mineral deposition. Our results agree with the studies from Stein and Lian, and Choi et al., who showed that the peak of ALP expression was observed during the post-proliferative period, and cells stained positive for ALP during the early mineralization stage, were followed by the decreased ALP expression during the heavy mineralization stage [43,44]. Assessment of the ALP expression at the mRNA level might show the peak expression at an earlier timepoint [47,48]. 

Recently, studies have highlighted the beneficial effect of ART and its derivatives on various stem cell lines towards cell survival and osteogenic differentiation. More specifically, dihydroartemisinin (DHA), an active compound within Artemisia annua, has been found to promote osteogenic differentiation of human mesenchymal stem cells (hMSCs), probably through the activation of ERK1/2 as well as Wnt/β-catenin pathways [16]. This was further supported by the study from Hu et al., where the exposure of dental pulp stem cells (DPSCs) to ART at the dose of 40 μM enhanced cell survival as well as osteogenic differentiation which was diminished by hypoxia and inflammation preconditioning [17]. The beneficial effects of ART administration on DPSCs seem to be related to the CA9-mediated antioxidant response and Wnt/β-catenin activation [17]. Incubation of bone marrow MSCs with ART following exposure to an ROS-induced environment resulted in increased cell survival, indicating an ART-mediated protection through the stimulation of the c-Raf-Erk1/2-p90rsk-CREB signaling pathway [18]. Moreover, ART compounds have been demonstrated to suppress osteoclast differentiation via the downregulation of pathways involved in RANKL-induced osteoclastogenesis [9].

Periodontitis is a disease characterized by an inflammatory process that leads to alveolar bone loss, and its progression involves interactions among inflammatory cells and ROS, highlighting the relationship between ROS production and periodontitis [49,50]. Oxidative stress affects bone remodeling, resulting in increased osteoclast activity and decreased osteoblast activity, causing a metabolic imbalance [51]. The therapeutic potential of ART is based on the ROS-induced toxicity that is caused by the parasite [4]. However, when administered in MSCs, ART exhibits cytoprotective properties, which involve a decrease in ROS [18]. hPDLCs incubation with ART and ART-loaded NPs with OM (ART-Si, ART-SiCa) led to a significant decrease in the antioxidant capacity after 21 days when compared with the control cells, whereas those cultured with the ART-SiCaCe1, ART-SiCaCe2.5 and ART-SiCaCe5 NPs maintained their antioxidant capacity throughout the culture period. The reduced antioxidant capacity directly affects the in vitro deposition of the calcium process, as it is obvious by the lack of a significant increase regarding mineral deposition between the Si/SiCa NPs and the ART-loaded Si/SiCa NPs. ART treatment has been found to exhibit cytoprotective and oxidoreductive properties via the activation of the ERK1/2-CREB-related pathway, that leads to the inhibition of caspase 3 and other related cell death enzymatic activities [18]. 

Regarding bioactivity assessment, HAp formation was observed on the surface of all studied materials before ART loading. Following ART loading, HAp formation appears to be rather constrained. The results on the antioxidant activity combined with the results from the bioactivity and the alizarine experiments indicate that the ART and cerium presence in the NPs have a synergistic role controlling the redox status and reducing the ROS in the hPDLCs, and, by extension, affect the in vitro deposition of the calcium process and HA formation. Increasing the Ce amount in the ART-loaded NPs inversely affected the mineral deposition process and HA formation by the hPDLCs. ART-loaded SiCaCe1 doped NPs emerged as the optimum condition, with both antioxidant and osteogenic properties, indicating that the low cerium amount combined with ART enhance the osteogenic differentiation of hPDLCs and the in vitro deposition of the calcium process. This is the first study to highlight the interplay between ART and Ce amount regarding the osteogenic differentiation of PDL cells and in vitro deposition of the calcium process, however, further studies should be performed to unravel the underlying pathways and the interactions between ART and Ce-NPs. Increased amounts of Ce in the presence of ART (in the case of ART-SiCaCe5) seem to neutralize each other, resulting in restricted hPDLCs osteogenic differentiation. Future experiments should be performed to assess gene expression regarding the osteogenic differentiation of hPDLCs, as well as antioxidant enzymes.

The results from this in vitro study seem promising for future application in periodontal tissue engineering. While it bears the inherent defects of an in vitro study, failing to replicate the conditions of cells in an organism, the in vivo application of ART-loaded SiCaCe NPs might provide more promising results, since the osteoprotective role of ART is also attributed to the inhibition of osteoclastic differentiation [9]. ART treatment can be beneficial in cases where bone regeneration is needed (bone defects), affecting the metabolic balance of bone deposition and resorption, as well as in cases of periodontal disease, where oxidative stress and inflammatory response leads to increased bone resorption. In detail, ART can suppress RANKL-induced osteoclastogenesis through the downregulation of various transcription factors, such as c-Fos, c-Jun, MITF and NF-κBand NFATc1. Additionally, ART can induce programmed cell death to osteoclasts through ferroptosis, an iron- and ROS-related form of programmed cell death [51]. Future research should focus on the cellular uptake of ART and in vivo studies evaluating the effects of the local application of ART in bone defects. 

## 5. Conclusions

Under the limitations of the present in vitro study, the following conclusions can be drawn:

1. Calcium silicate NPs doped with Ce can induce osteogenic differentiation of hPDLCs and in vitro deposition of calcium;
2. The presence of ART can promote the osteogenic differentiation of hPDLCs;
3. Increasing the Ce amount in the ART-loaded NPs can inversely affect the mineral deposition process by the hPDLCs and HA formation;
4. ART and Ce in the NPs have a synergistic role controlling the redox status and reducing the ROS in the hPDLCs, affecting the in vitro deposition of the calcium process and HA formation.

Overall, the results of this study suggest that by modulating the concentration of cerium and ART on cerium-doped Si-Ca NPs, promising materials can be developed, with possible applications as fillers in regenerative membranes, hydrogels and scaffolds towards periodontal and bone tissue regeneration.

## Figures and Tables

**Table 1:** Nominal compositions of synthesized mesoporous NPs in %mol.

Sample	SiO_2_	CaO	CeO
Si	100.0		
SiCa	60.0	40.0	
SiCaCe1	60.0	39.0	1.0
SiCaCe2.5	60.0	37.5	2.5
SiCaCe5	60.0	35.0	5.0

## Data Availability

All data are presented in the manuscript.
